# Multi-Entropy Feature Concatenation for Data-Efficient Cross-Subject Classification of Alzheimer’s Disease and Frontotemporal Dementia from Single-Channel EEG

**DOI:** 10.3390/e28020212

**Published:** 2026-02-12

**Authors:** Jiawen Li, Chen Ling, Weidong Zhang, Jujian Lv, Xianglei Hu, Kaihan Lin, Jun Yuan, Shuang Zhang, Rongjun Chen

**Affiliations:** 1 School of Computer Science, Guangdong Polytechnic Normal University, Guangzhou 510665, China; lijiawen@gpnu.edu.cn (J.L.); lingchen@gpnu.edu.cn (C.L.); zwd@stu.gpnu.edu.cn (W.Z.); jujianlv@gpnu.edu.cn (J.L.); huxianglei@gpnu.edu.cn (X.H.); kaihanlin@gpnu.edu.cn (K.L.); yuanjun@gpnu.edu.cn (J.Y.); 2 State Key Laboratory for Novel Software Technology, Nanjing University, Nanjing 210023, China; 3 ZUMRI-LYG Joint Laboratory, Zhuhai UM Science and Technology Research Institute, Zhuhai 519031, China; 4 School of Artificial Intelligence, Neijiang Normal University, Neijiang 641004, China; 5 School of Life Science and Technology, University of Electronic Science and Technology of China, Chengdu 610056, China; 6 Guangdong Provincial Key Laboratory of Intellectual Property and Big Data, Guangdong Polytechnic Normal University, Guangzhou 510665, China

**Keywords:** electroencephalography (EEG), entropy feature concatenation, Alzheimer’s disease (AD), frontotemporal dementia (FTD), biomedical engineering

## Abstract

Alzheimer’s disease (AD) and frontotemporal dementia (FTD) are neurodegenerative disorders where early detection is vital. However, the need for long-term monitoring is incompatible with data-scarce settings, and methods trained on one subject often fail on another due to cross-subject variability. To address these limitations, this study proposes a cross-subject, single-channel electroencephalography (EEG)-based method that uses Multi-Entropy Feature Concatenation (MEFC) to classify AD and FTD. First, single-channel EEG is processed through the Discrete Wavelet Transform (DWT) to extract five rhythms: delta, theta, alpha, beta, and gamma. Subsequently, Permutation Entropy (PE), Singular Spectrum Entropy (SSE), and Sample Entropy (SE) are calculated for each rhythm and concatenated to form a combined MEFC to characterize the non-linear dynamic properties of EEG. Lastly, Dynamic Time Warping (DTW), Pearson Correlation Coefficient (PCC), Wavelet Coherence (WC), and Hilbert Transform Correlation (HTC) are employed to measure the similarity between unknown rhythmic MEFC and those from AD, FTD, and Healthy Control (HC) groups, performing a data-driven classification via similarity measurement. Experimental results on 88 subjects in the AHEPA dataset demonstrate that the beta-rhythm with PCC yields a three-class accuracy of 76.14% using single-channel FP2. In another dataset, the Florida-Based dataset, involving 48 subjects, theta-rhythm with WC achieves a two-class accuracy of 83.33% using FP2. Furthermore, a MATLAB R2023b-based toolbox is developed using the proposed method. Such outcomes are impressive, given the limited data per individual (data-efficient), reliable performance across new subjects (cross-subject), and compatibility with wearable devices (single-channel), providing a novel entropy-based approach for EEG-based applications in biomedical engineering.

## 1. Introduction

Memory is a fundamental cognitive function, essential for comprehending the outside environment, constructing personal identity, and facilitating reasoning and prediction. An individual’s behavior and social interactions are profoundly influenced by their memory. Nevertheless, Alzheimer’s disease (AD) progressively erodes this function, leading to the impairment of communication abilities, a growing dependence on around-the-clock care, and the severe deterioration of physical functions. This decline imposes considerable psychological and emotional strain on patients and a substantial burden on their families. Currently, approximately 55 million people live with dementia worldwide, a figure projected to rise to 139 million by 2050, with AD accounting for the majority of these cases [1].

Frontotemporal dementia (FTD) is another neurodegenerative disorder distinct from AD, characterized by its impact on the frontal and temporal lobes, which are central to personality, behavior, language, and executive functions [2]. Consequently, patients typically present with symptoms such as disinhibition, apathy, loss of empathy, and executive dysfunction. As the clinical manifestations of both AD and FTD can overlap with normal aging or other neurological conditions, existing diagnostic approaches are susceptible to misdiagnosis, frequently resulting in a critical delay in intervention. Although no cure currently exists for either neurodegenerative condition, early treatment can slow disease progression, which highlights the urgent need for long-term monitoring and automated classification methods to mitigate patient suffering and help with daily living activities [3].

Electroencephalography (EEG) is a non-invasive technique that records the brain’s electrical activity via electrodes placed on the scalp. Compared to techniques with higher operational demands, such as Magnetic Resonance Imaging (MRI) and Positron Emission Tomography (PET) [4] or invasive methods like Electrocorticography (ECoG) [5], EEG offers advantages including high temporal resolution and the ability to collect complex neural dynamics in real time. Furthermore, current EEG-based systems are relatively portable and cost-effective, making them suitable for supporting the clinical assessment of neurodegenerative disorders such as AD and FTD.

Previously, several EEG-based approaches have been proposed in this field. For example, Sharma et al. [6] integrated graph-theoretical and statistical features within a Convolutional Neural Network (CNN) model, achieving 99.48% accuracy for AD. Yu et al. [7] combined EEG signals with genotype data and polygenic risk scores to train a Support Vector Machine (SVM) classifier, achieving an accuracy of approximately 92% for AD detection. Using resting-state EEG, Nour et al. [8] presented a hybrid model based on Deep Ensemble Learning (DEL) and a 2D-CNN, attaining 97.9% accuracy for AD detection in a five-fold cross-validation. Jain et al. [9] employed a synchro-squeezing transform followed by a fine-tuned pre-trained CNN, achieving 98.50% accuracy for AD detection using the P3 channel. Further advancements in deep learning are demonstrated by Zhang et al. [10], who developed an end-to-end deep neural network that enhances classification accuracy. Also, Shen et al. [11] designed a deep learning model based on multi-band Morlet mutual information functional connectivity to detect FTD, which attains an accuracy of 90.38%.

The above studies indicate the potential of EEG, particularly when applied with machine learning classifiers or deep learning models, for the automated detection of AD and FTD. However, translating these findings into practical healthcare applications for daily use faces three interconnected challenges. First, data limitations make it difficult to obtain extensive, labeled EEG recordings from patient populations, reducing the efficiency of deep learning model training. Second is poor cross-subject generalization, where classification methods trained on one subject usually fail on new individuals due to high individual variability. The third is the need to minimize the use of portable devices toward single-channel setups to ensure patient comfort and suitability for long-term, wearable monitoring. Therefore, a solution that addresses these three constraints, i.e., operating under data-limited conditions, providing cross-subject generalization, and using a single-channel EEG, for reliable classification of AD, FTD, and Healthy Control (HC), is meaningful.

To bridge this gap, this study proposes a novel EEG-based classification method via Multi-Entropy Feature Concatenation (MEFC). Its innovation extracts and concatenates complementary entropy features to maximize information yield from minimal single-channel data, thereby enhancing method stability and generalizability across subjects. By achieving data-efficient classification under these constraints, a meaningful step toward the design of easy-to-use EEG-based healthcare aids for managing neurodegenerative disorders is presented.

**Figure 1:**
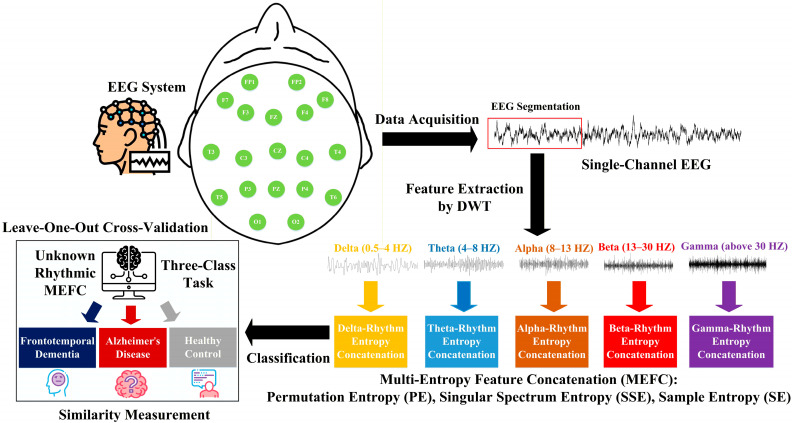
The overall framework of the proposed method for classifying AD and FTD via MEFC from a single-channel EEG.

To this end, the representative entropies that are closely associated with the psychophysiological complexity of AD and FTD within EEG signals, i.e., Permutation Entropy (PE) [12], Singular Spectrum Entropy (SSE) [13], and Sample Entropy (SE) [14], are obtained, which comprehensively provide the characterization of signal irregularity and dynamic variation for enhancing classification performances under single-channel condition. Specifically, PE quantifies the predictability of local ordinal patterns in the EEG to characterize the degree of neural activity disorder along the temporal dimension. It helps discriminate subtle differences in neuroelectrical dynamics among AD, FTD, and HC groups by providing temporally sensitive details for classification. SSE describes the intrinsic structural complexity of a signal and the uniformity of its energy distribution in the frequency domain. It reflects stability in oscillatory brain activity and assesses the differential impact of dementia subtypes on brain rhythm structure. SE reveals the overall irregularity and self-similarity of EEG, and it is usually decreased in AD patients compared with HCs, making it a biomarker of altered brain activity in AD. Based on this, these entropies are extracted and concatenated to form a combined MEFC specific to five brain rhythms, delta (0.5–4 Hz), theta (4–8 Hz), alpha (8–13 Hz), beta (13–30 Hz), and gamma (above 30 Hz) [15], which serve as the feature vector for the classification via similarity measurement between the unknown rhythmic MEFC and those of AD, FTD, and HC groups. Technically, a higher similarity indicates greater information overlap, indicating they are in the same group, while a lower similarity reveals they are less likely to be the same. Since the rhythmic MEFC includes details on brain rhythms and entropies, a high similarity value can be interpreted as a similar brain state, providing a data-driven classification based on entropy analysis. Unlike deep learning models, this method does not rely on large amounts of data for training, making it resource-efficient for single-channel EEG. Hence, by evaluating all channels along with specific rhythm, the channel that provides an in-depth understanding of how the rhythmic MEFC can be effectively improved for classifying cross-subject AD and FTD under data-limited conditions is identified. Additionally, a toolbox based on the proposed method is implemented. For better illustration, the overall framework is depicted in Figure 1, and the contributions of this study are summarized as follows:

1. To address data limitations, a novel classification method based on MEFC and similarity measurement is proposed, which extracts and leverages entropy features from limited EEG samples per subject, reducing dependence on large-scale datasets for training complex models.
2. To support cross-subject generalization, the channel selection is incorporated that identifies the most discriminative single channel, yielding the highest cross-subject classification accuracy. This process not only validates the physiological relevance of the channel for AD and FTD but also enhances the applicability across different individuals by focusing on stable single-channel EEG.
3. To achieve a practical application, a toolbox based on the proposed method is provided. It enables three-class (AD, FTD, HC) classification from single-channel EEG, facilitating the development of portable, user-friendly devices suitable for long-term monitoring of such neurodegenerative disorders in daily living.

The remainder of this paper is organized as follows: Section 2 details the experimental datasets (AHEPA and Florida-Based). The proposed method is presented in Section 3, including feature extraction, MEFC, and classification via similarity measurements. Section 4 shows statistical results and toolbox implementation, along with the comparative study and method discussion. Finally, Section 5 provides the conclusions and future prospects.

## 2. Experimental Datasets

The first experimental dataset is a publicly accessible dataset collected by the second Department of Neurology at the AHEPA University Hospital in Thessaloniki, Greece [16]. Its key parameters are listed in Table 1, comprising 88 subjects: 36 patients with AD, 23 with FTD, and 29 HCs. Their mean ages are 66.4, 63.6, and 67.9 years for AD, FTD, and HC, respectively. For each subject, resting-state EEG data were recorded employing a 19-channel system at 500 Hz, with two reference electrodes (A1, A2) placed on the mastoid process. The average recording durations are approximately 13.5, 12.0, and 13.8 min for AD, FTD, and HC, respectively.

The second experimental dataset was gathered by researchers from Florida State University [17]. It includes two groups, differentiated by recording condition and participant type: one group consists of 24 HC elderly individuals, while the other has 24 patients diagnosed with AD. EEG data were recorded using a 19-channel system at 128 Hz, with an 8 s recording duration in both cases.

**Figure 2:**
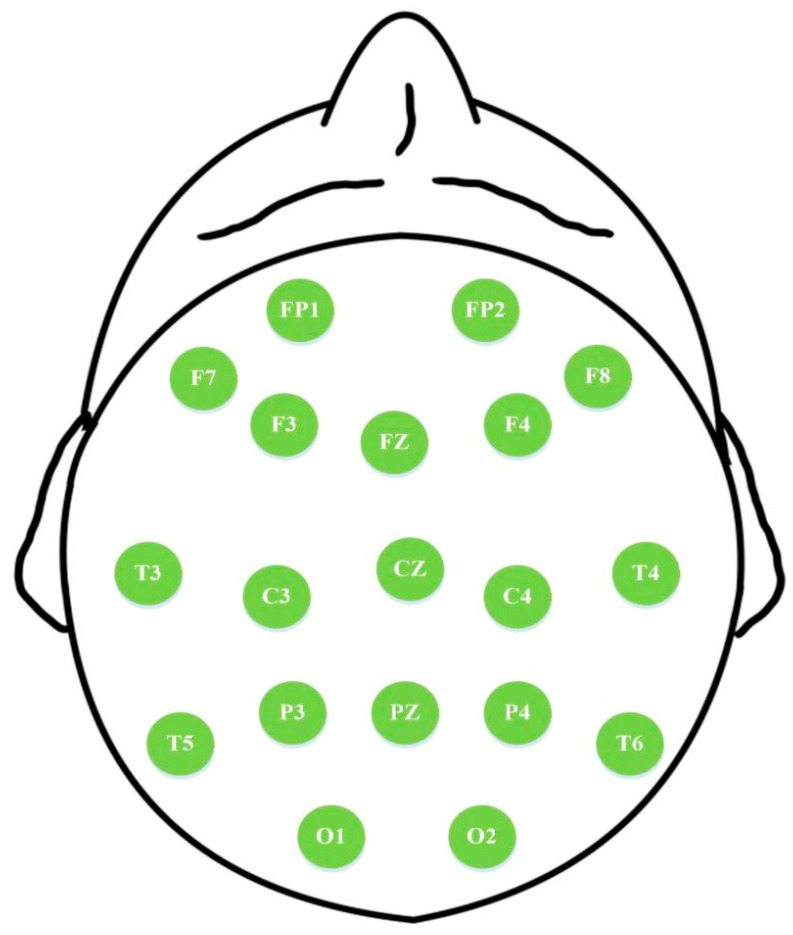
The 19-channel EEG system of the experimental datasets.

The spatial arrangement of the 19 EEG channels in the AHEPA and Florida-Based dataset, following the international 10–20 system, is illustrated in Figure 2, enabling the mapping of MEFC differences to specific scalp locations and facilitating channel selection for classification. Therefore, it establishes an essential basis for classifying AD and FTD from an entropy-driven EEG-based approach. Furthermore, these datasets present characteristically data-limited scenarios, which align with the practical constraints to be addressed in this study. First, the total number of subjects and the per-class sample sizes are relatively small for training complex deep learning models. Second, the recording period per subject imposes a strict upper limit on the usable signal obtainable from each individual. Most critically, in a Leave-One-Out Cross-Validation (LOOCV) scheme, the classification method must learn to accurately classify a new individual based on patterns derived from all others, making the effective training data for each validation fold inherently scarce. Hence, these datasets are appropriate for this study.

## 3. Proposed Method

### 3.1. Feature Extraction

In this study, the entire 8 s EEG for each subject in the Florida-Based dataset is analyzed. However, for the AHEPA dataset, since the durations vary across groups, to mitigate redundancy in EEG recordings and ensure uniform data input for classification, a 30 s segment is extracted from each signal. This specific duration was determined through empirical pilot analyses that compared the performance of features extracted from 10 s, 20 s, and 30 s segments. The 30 s segment consistently yielded the most stable and discriminative features for classification, balancing information sufficiency and computational efficiency. Critically, for all subjects in the AHEPA dataset, the first 30 s of the recording immediately after the start of the resting-state or task period are selected, ensuring consistency across the entire dataset and eliminating any subjective bias in data segment selection.

Subsequently, these EEG segments from two datasets are decomposed using the Discrete Wavelet Transform (DWT) to separate five classical brain rhythms. DWT is well-suited for analyzing non-stationary, non-linear signals such as EEG, as it simultaneously decomposes a signal into high-frequency and low-frequency coefficients at diverse scales [18]. The multi-level decomposition facilitates the separation of rhythmic details associated with distinct states. Mathematically, the decompositions via DWT are defined by Equations (1) and (2):

(1) $$A_{j+1}\left[k\right]=\sum_{n}h\left[n-2k\right]A_{j}[n]$$

(2) $$D_{j+1}[k]=\sum_{n}g[n-2k]D_{j}[n]$$

where *A_j_* represents the approximate coefficient of the *j*-th layer, *D_j_* denotes the detail coefficient of the *j*-th layer, *h*[*n*] means the low-pass filter, *g*[*n*] refers to the high-pass filter, *n* is the index of the discrete sampling point of the current layer signal, and *k* is the index of the wavelet coefficient after down-sampling.

**Figure 3:**
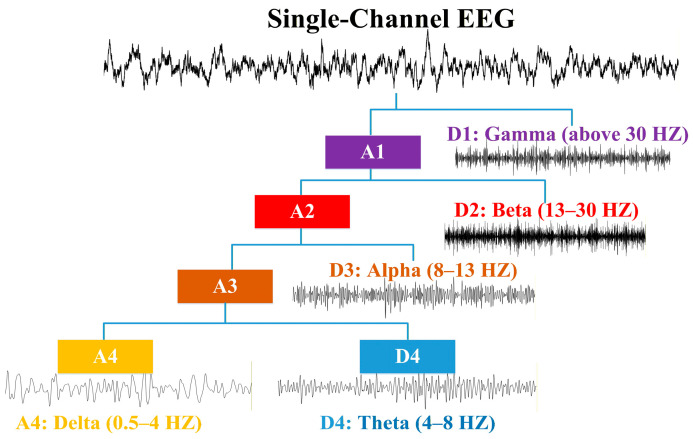
Multi-level decomposition of the five rhythms from an EEG signal using a 4-level DWT.

The Daubechies 4 (db4) wavelet is selected as the basis function for the DWT due to its adequate smoothness and sufficient vanishing moments, providing a trade-off between time and frequency localization [19]. Specifically, db4 is known to offer a practical compromise. Its relatively short support length provides good temporal resolution to capture transient events in EEG, while its number of vanishing moments is sufficient to yield a frequency response sharp enough to reasonably isolate the standard EEG rhythm bands (delta, theta, alpha, beta, and gamma). These characteristics make it suitable for analyzing EEG. In this study, EEG signals from the AHEPA dataset are down-sampled to 128 Hz, while those from the Florida-Based dataset are already 128 Hz. Hence, they can be decomposed using a four-level DWT to separate five brain rhythms, as depicted in Figure 3.

After that, PE, SSE, and SE are obtained for each brain rhythm. PE quantifies signal regularity by assessing the frequency of specific ordinal patterns, with higher values indicating complex brain dynamics and lower values reflecting pathological regularity. Its critical importance lies in its sensitivity to the distinct network disruptions caused by AD and FTD [20]. AD, associated with posterior pathology, and FTD, characterized by frontal temporal degeneration, differentially impair neural synchrony. Neurologically, a decrease indicates AD, whereas a more focal reduction in frontal regions could imply FTD. Its calculation is given by the following Equation:

(3) $$PE=-\sum_{j=1}^{k}p(\pi_{j})\times lnp(\pi_{j})$$

where *p*(*π_j_*) represents the probability of occurrence of the *j*-th ordinal pattern *π_j_*, which is derived by comparing the relative amplitude order of data points within a segment; it constitutes a form of order-based symbolic discretization, where the continuous-valued signal is transformed into a sequence of discrete symbols based solely on the local rank order.

The SSE quantifies the signal’s temporal structure by assessing the energy distribution across its principal orthogonal components. Derived from Singular Spectrum Analysis (SSA), SSE decomposes the time-domain signal via embedding and Singular Value Decomposition (SVD) into a set of orthogonal components. The squared singular values represent the energy contributed by each component, and the normalized energy distribution forms a probability vector, whose Shannon entropy is computed as the SSE. Particularly, this energy refers to the variance in the embedding space, and the components are data-driven, orthogonal temporal modes. Usually, a highly concentrated energy distribution (low SSE) indicates dominance by a few, possibly pathological, rhythmic patterns. In contrast, a more balanced, distributed energy profile (higher SSE) implies the healthy, collaborative engagement of multiple neural oscillators and functional networks. That means the importance of SSE in classifying AD and FTD lies in its reflection of large-scale network integration and synergy, which is fundamentally disrupted in these dementias [21]. As a result, SSE represents the resulting collapse of synergistic complexity due to an impaired brain network, expressed as follows:

(4) $$\rho_{i}=\frac{\sigma_{i}^{2}}{\sum_{i-1}^{L}\sigma_{i}^{2}}$$

(5) $$SSE=-\sum_{i=1}^{L}\rho_{i}ln\rho_{i}$$

where *ρ_i_* denotes the percentage of the energy of the *i*-th component in the total energy of the signal, and *σ_i_* refers to the *i*-th singular value, which represents the intensity of the *i*-th component of the signal.

SE indicates the conditional probability that similar sequences of data points remain similar at the next point, making it sensitive to the inherent unpredictability of time-series data. Its higher value denotes greater irregularity and complexity, implying a healthy neural system capable of generating dynamic patterns, while its lower value reflects a loss of complex variability, which can be regarded as a biomarker of a pathologically constrained system. As a result, the critical insight SE provides in the context of AD and FTD lies in its sensitivity to the early, subtle disintegration of neural dynamics driven by synaptic failure [22]. The progressive neuronal loss in these dementias leads to a fundamental simplification of brain electrophysiology. Once complex and adaptive EEG activity becomes more periodic and stereotyped. SE excels at quantifying this variation from a complex state to a regular one, making it appropriate for the classification task in this study:

(6) $$SE(m,r)=-ln(\frac{\varphi (m,r)}{\varphi (m+1,r)})$$

where *m* denotes the embedding dimension, which is fixed at two in this study, *r* refers to the control threshold, defined as *r* = 0.15 × *σ* (*σ* is the standard deviation of the time series being analyzed), and *φ*(*m*, *r*) means the count of statistically similar vector pairs. Similarity is assessed using the Chebyshev distance, where two embedding vectors are considered similar if the maximum absolute difference between all their corresponding components is less than or equal to *r*.

### 3.2. Multi-Entropy Feature Concatenation (MEFC)

The MEFC integrates the representative entropies relevant to the pathophysiology of AD and FTD, providing comprehensive details for characterizing alterations in brain complexity. This feature concatenation is of considerable value for EEG-based classification of neurodegenerative diseases, as it leverages the complementary strengths of diverse entropies. As stated, SSE reveals the structural complexity and temporal stability of brain activity patterns. PE is sensitive to non-linear dynamics and the predictability of signal patterns, both of which are beneficial for detecting subtle alterations in brain state. SE quantifies signal irregularity and self-similarity, reflecting the complexity of the underlying neural processes. By combining them into a rhythm-based feature vector, the MEFC enables interpretation of EEGs associated with neurodegeneration. Therefore, the proposed method employs entropy-driven features to generate a rhythm-specific MEFC for each channel. That is, for each of the five rhythms (delta, theta, alpha, beta, and gamma), a distinct MEFC vector is constructed and subsequently used as an independent input to the classifier, facilitating a direct comparison of their discriminatory power. The MEFC for a given channel and rhythm is denoted as follows:

(7) $${MEFC}_{Rhythm}=[{PE}_{Rhythm}\;{SE}_{Rhythm}\;{SSE}_{Rhythm}]$$

The MEFC is advantageous for addressing challenges of data scarcity, cross-subject variability, and single-channel settings. In data-limited conditions, concatenating vital entropies creates an information-dense feature representation from minimal data. By capturing fundamental and complementary aspects of signal complexity that are more likely to be invariant across individuals, the MEFC enhances cross-subject generalization, reducing dependence on individual-specific data. Additionally, the entropy feature vector’s computational cost and low dimensionality are well-suited to single-channel EEG-based applications, which are valuable for deployment on portable devices.

### 3.3. Classification Method

In this study, classification is performed by measuring the similarity between the rhythmic MEFC of an unknown sample and those of AD, FTD, and HC groups. For instance, an unknown MEFC is compared with MEFCs from AD, FTD, and HC to obtain respective similarity levels. If the unknown MEFC shows the highest level of similarity with the AD, it indicates that this testing sample can be classified into the AD group. Measuring similarity among diverse brain states for classification is reasonable, as the MEFC contains the pathological entropy variation. Furthermore, this similarity-based classification method provides a lightweight solution for data-limited conditions. By comparing an unknown MEFC to prototypical group profiles, it discerns whether the entropy pattern more closely resembles the posterior complexity loss in AD, the anterior-focused dysregulation in FTD, or the balanced profile of HC. Consequently, the classification method not only leverages the sensitivity of multi-entropy features but also solidifies the analysis of entropy in EEG-based studies, even with limited data for cross-subject model training. To this end, four typical similarity measurements are investigated: Dynamic Time Warping (DTW), Pearson Correlation Coefficient (PCC), Wavelet Coherence (WC), and Hilbert Transform Correlation (HTC).

DTW excels where traditional Euclidean distance fails, specifically, when data vary in length or exhibit phase shifts and local accelerations. Its core algorithm non-linearly warps the time axis to find a minimal-distance alignment path between data, effectively comparing their overall shape rather than their point-for-point correspondence. For classifying AD and FTD using MEFC, physiological and cognitive variability inevitably introduces temporal misalignments between subjects’ entropy profiles. Therefore, DTW allows for elastic matching, which can align a rapid entropy fluctuation in one state with a more protracted, yet morphologically similar fluctuation in another:

(8) $$D\left(i,j\right)=dist\left(x_{i}+y_{j}\right)+min\left\{D\left(i-1,j\right),D\left(i,j-1\right),D\left(i-1,j-1\right)\right\}$$

where *D*(*i*, *j*) represents the element at (*i*, *j*) in the cumulative distance matrix, while *dist*(*x_i_*, *y_j_*) denotes the local distance function, which calculates the difference between two datasets at specific time points, embodying the DWT’s minimal cumulative path selection.

PCC assesses the strength of a linear relationship between two feature vectors by measuring their covariance normalized by the product of their standard deviations. This parametric measure is effective for normally distributed data and provides a direct interpretation of linear associations in brain network complexity features. When applied to the MEFC, PCC quantifies the linear functional connectivity or disconnection at the system-level coordination of complexity:

(9) $$PCC(X,Y)=\frac{\sum_{i=1}^{n}\left(X_{i}-\bar{X}\right)\left(Y_{i}-\bar{Y}\right)}{\sqrt{\sum_{i=1}^{n}{\left(X_{i}-\bar{X}\right)}^{2}}\sqrt{\sum_{i=1}^{n}{\left(Y_{i}-\bar{Y}\right)}^{2}}}$$

where *X* and *Y* are two feature vectors, *n* is the number of data points, $\bar{X}$ and $\bar{Y}$ are the means of *X* and *Y*, respectively, and *X_i_* and *Y_i_* are the *i*-th elements of each vector. The PCC value ranges from −1 (negative linear correlation) to +1 (positive linear correlation), with absolute values indicating the strength of the linear correlation.

WC measures the local correlation between two non-stationary data simultaneously in the time and frequency domains. It reveals the dynamic coupling strength and phase relationships across different oscillatory frequencies and specific time points, showing the temporally fragile, frequency-specific disintegration of functional networks. The neurodegenerative processes in AD and FTD progressively degrade the capacity for flexible, task-dependent coupling. In this regard, WC can pinpoint these deficits via a multi-scale similarity measurement of brain disorders:

(10) $$R_{n}^{2}\left(s\right)=\frac{{\left|S\left(s^{-1}W_{n}^{XY}(s)\right)\right|}^{2}}{S(s^{-1}{\left|W_{n}^{X}(s)\right|}^{2})\times S(s^{-1}{\left|W_{n}^{Y}(s)\right|}^{2})}$$

where *s* represents the scale, which is inversely proportional to frequency (or period) in wavelet analysis, $W_{n}^{X}(s)$ and $W_{n}^{Y}(s)$ are the Continuous Wavelet Transform (CWT) coefficients of *X* and *Y*, respectively. These coefficients reflect the energy and phase of the original signals at specific times and scales. $W_{n}^{XY}(s)$ is the cross-wavelet transform coefficient, and *s*^−1^ denotes the scale normalization factor that corrects for energy across different scales to ensure consistent results. In this study, the CWT is computed using a complex Morlet wavelet (cmor1–1.5) with scales ranging from 1 to 10 in steps of 0.5. The smoothing operator *S* is applied as an arithmetic average over the entire time dimension *n*, yielding a scale-dependent measure of average coherence across the analysis epoch. This approach provides a global estimate of Wavelet Coherence while balancing the wavelet’s inherent trade-off between time-frequency resolution: higher frequencies offer better temporal localization, while lower frequencies provide better frequency resolution.

HTC calculates the instantaneous phase employing the Hilbert Transform (HHT) and a phase synchrony index that quantifies the consistency of the phase relationship between two entropy trajectories. It is not concerned with the absolute magnitude of entropy values. However, it mainly focuses on measuring the similarity in their temporal modulation patterns, essentially, whether the rises and falls in EEG signals are consistently aligned with those in another over time. Please note that this study does not apply the HHT to the raw EEG signal itself. Instead, the transform is applied to the derived entropy feature time series to obtain their analytic signals. The similarity level of HTC is then computed as the correlation between the instantaneous envelopes of these signals:

(11) $$H_{\alpha }\left\{x[n]\right\}=x[n]+H\left\{x[n]\right\}$$

(12) $$HTC(x_{1},x_{2})=\left|\rho (\left|H_{\alpha }\left\{x_{1}[n]\right\}\right|,\left|H_{\alpha }\left\{x_{2}[n]\right\}\right|)\right|$$

where *H*{} refers to the HHT operator and *ρ*(*X*, *Y*) represents the PCC.

Furthermore, a similarity-based classification method needs a template for each class as a basis. To account for cross-subject variability and maximize the use of available data, template selection is performed based on LOOCV. In each fold, the mean of the rhythmic MEFCs of all FTD samples in the training set defines the FTD template. Similarly, the mean of those MEFCs for the AD and HC samples constitutes their respective templates. The left-out sample serves as the unknown test data, and its MEFC is compared against the three templates for classification employing the four similarity measurements presented above. This process is repeated until each sample has been used once as unknown test data. In this study, the performance is mainly evaluated using accuracy:

(13) $$Accuracy=\frac{TN+TP}{TP+TN+FN+FP}$$

where True Positive (TP) represents samples correctly identified as belonging to that class, False Positive (FP) denotes samples incorrectly assigned to the target class (they belong to a different class), False Negative (FN) means samples from the target class that are incorrectly assigned to another class, and True Negative (TN) refers to the samples correctly identified as not belonging to the target class. The overall accuracy is calculated from the confusion matrix as the total number of correctly classified instances (sum of the diagonal) divided by the total number of samples.

## 4. Results and Discussion

### 4.1. Statistical Results

In this study, the experiment was implemented via MATLAB R2023b, and to ensure reproducibility of the results and the use of the toolbox within the research community, the MATLAB code is available on “https://github.com/LC20251214/AD-and-FTD.git (accessed on 22 December 2025)”. Then, a data-driven statistical analysis of the experimental results was performed, yielding insights into the classification of AD and FTD across various EEG channels.

**Figure 4:**
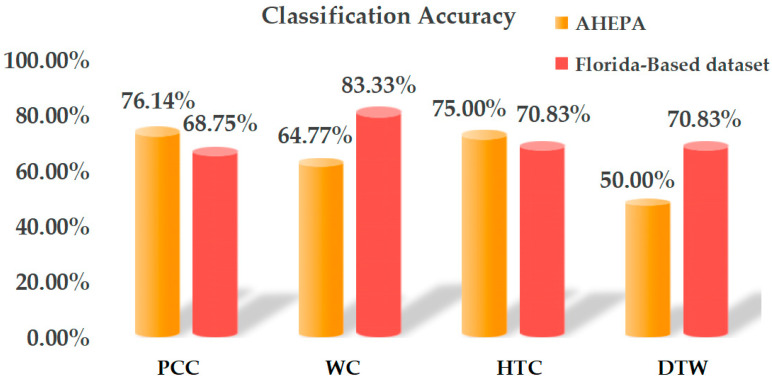
The highest classification accuracies across different similarity measurements for classifying MEFCs using single-channel EEG data (FP2).

After testing four similarity measurements and the five rhythmic MEFCs across 19 channels, as shown in Figure 4, PCC, WC, HTC, and DTW showed differences in the same channel with the highest accuracy. Regarding the three-class task on the AHEPA dataset, the highest accuracies were 76.14%, 64.77%, 75.00%, and 50.00%, respectively, while in the two-class task on the Florida-Based dataset, the highest accuracies were 68.75%, 83.33%, 70.83%, and 70.83%, respectively. Note that these results are all derived from the MEFCs of the FP2 channel. Meanwhile, the inter-channel stability analysis, measured by the Mean Absolute Deviation (MAD) of classification accuracy, revealed that the MAD values for PCC, WC, HTC, and DTW were 5.23%, 5.49%, 5.28%, and 6.07% in the three-class task, respectively, and 5.89%, 7.91%, 6.05%, and 9.14% in the two-class task. These results indicate that PCC and WC perform well with single-channel EEG, suggesting their suitability in the proposed method.

Next, to identify the most promising brain rhythm for classifying AD and FTD in this study, the performance of five rhythmic MEFCs was analyzed from two key perspectives. One was the highest classification accuracy, which revealed the upper limit of the classification’s discriminative capability. Another was stability, which quantified fluctuations in accuracy across different channels. The results are listed in Table 2.

Table 2 demonstrates that in the three-class task, the single-channel classification accuracy of beta-rhythm MEFC is 76.14%, indicating that beta-rhythm oscillatory activity encodes specific neural information related to the pathology of AD and FTD. Regarding the two-class task, the single-channel classification accuracy of theta-rhythm MEFC is 83.33%, suggesting that theta-rhythm oscillatory activity exhibits pronounced neural characteristics associated with AD pathology. Additionally, the beta-rhythm MAD is 3.91% in the three-class task, and the MADs of theta-rhythm is 8.44% in the two-class task, respectively. Such relatively low volatility indicates that beta-rhythm and theta-rhythm MEFC exhibit stable classification performance in AD and FTD.

Moreover, this study identifies a representative channel for distinguishing between AD and FTD in a three-class classification task, using the PCC similarity-based classification method and beta-rhythm MEFC. The results show that the FP2 channel achieves the highest accuracy of 76.14% in cross-subject single-channel classification, indicating that the prefrontal region carries pathophysiological information relevant to both AD and FTD when PCC is used as the classification method. As for the two-class task of distinguishing AD from HC using WC and theta-rhythm MEFC, the FP2 channel conducts 83.33% classification accuracy, further verifying that the FP2 channel contains pathophysiological features associated with AD. Thus, developing a lightweight method that relies on the FP2 channel has a low computational cost and maintains a satisfying level of discriminative ability, promoting its implementation on portable devices for long-term healthcare monitoring.

### 4.2. Toolbox Implementation

**Figure 5:**
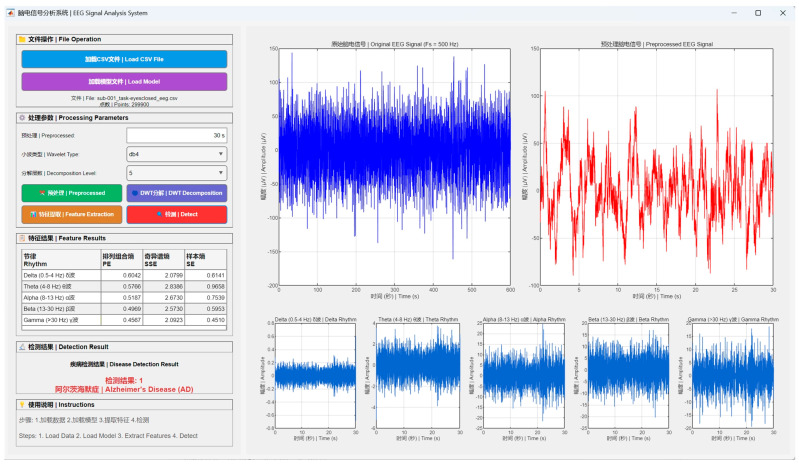
A MATLAB R2023b-based toolbox designed by the proposed MEFC method for classifying AD, FTD, or HC, with multilingual captions (English and Chinese).

To benefit the research community, a MATLAB-based toolbox is implemented, as shown in Figure 5. In four main steps (load data, load model, extract features, and detect), it handles single-channel EEG input (e.g., FP2 channel). Data processing employs the db4 wavelet DWT at multiple decomposition levels, after which five brain rhythms are extracted. For each rhythm, the toolbox acquires PE, SSE, and SE, which are then formed into the MEFC. A PCC-based classification via similarity measurement yields detection results (either AD, FTD, or HC, see Figure 5 for a sample result of AD). Moreover, the user-friendly Graphical User Interface (GUI) with multilingual captions (English and Chinese) makes it easy for neuroscientists, engineers, or clinicians without extensive programming experience. It also exhibits potential for integration into a single-channel device, extending entropy analysis to EEG-based work in biomedical engineering.

### 4.3. Comparative Study

To demonstrate the advantages of the proposed method, a comparative study is conducted against recent EEG-based works, as outlined in Table 3. 

This study addresses a gap in cross-subject EEG-based classification of AD and FTD by using a single-channel FP2. Previous studies have predominantly relied on multi-channel data with feature extraction to enhance accuracy, often overlooking the potential of single-channel paradigms. For instance, Zikereya et al. [24] extracted features from all channels for a Deep Adaptive Clustering (DAC) model, offering accuracies of 83.00% for AD vs. HC, 70.00% for FTD vs. HC, and 60.00% for FTD vs. AD. Dharia et al. [27] selected the six best-performing channels. Then they employed a Dual-Transformer Cross-Attention Framework (DTCA-Net) to achieve 85.20% classification accuracy for AD vs. HC and 67.70% for FTD vs. HC. Although the proposed method is not the most accurate, it offers a trade-off between the number of channels used and overall performance, achieving 76.14% three-class accuracy and 83.33% two-class accuracy with only a single-channel EEG. Consequently, its advantage lies in reducing data redundancy, enhancing the practicality of portable devices, and improving the feasibility of long-term monitoring, compared with state-of-the-art works.

Additionally, unlike previous studies that mostly employ complex deep learning architectures, this study uses a data-efficient similarity-based classification method. PCC and WC are chosen in this study because they are based on the mathematical principle of similarity to measure the co-change in signals, are sensitive to non-stationary signals, and are highly responsive to the inherent time patterns of entropy features. Considering the constraints of a data-limited dataset and the challenge of cross-subject variability, the performance is impressive, offering a new solution for classifying the neurodegenerative diseases AD and FTD from a single-channel EEG.

### 4.4. Discussion

First, this study demonstrates stable performance across two experimental datasets, providing preliminary validation of its generalization. It indicates that the entropy features extracted by the MEFC capture discriminative differences between AD and FTD. Meanwhile, this method exhibits lightweight characteristics. At the data level, it analyzes single-channel EEG signals, reducing dependence on multi-channel devices and complex spatial information processing. At the feature level, it extracts three entropy values from single rhythms to form low-dimensional feature vectors, thereby enabling efficient learning with limited data, and enhancing its practical potential in clinical small-sample scenarios.

Second, a similarity-learning framework is developed based on template matching. The key steps are extracting feature templates from the training set that best represent the AD and FTD categories, and classifying unknown samples based on the similarity measurement between these templates and the unknown samples within the learned metric space. The effectiveness of this framework relies entirely on the quality of the constructed templates, which themselves are defined by the full set of MEFC. In this regard, removing any one entropy feature would directly alter the physical definition of the templates, and any resulting change in performance reflects a loss of informational completeness in the templates, rather than the failure of an independent component. By integrating these three entropies, MEFC leverages their respective strengths, achieving impressive classification accuracy even under conditions of limited data and small sample sizes.

Third, the beta-rhythm MEFC distinguishes between AD, FTD, and HC in the three-class task, which is attributed to the increased connectivity between AD and FTD in the posterior frontal beta activity [30]. In the two-class task, theta rhythm effectively differentiates AD from HC, as AD patients typically exhibit abnormal theta-band activity characterized by elevated power and pathological reorganization of neural connectivity [31]. This finding properly explains why the theta rhythm achieves the highest classification accuracy in this study. These results align with existing works reporting consistent oscillatory alterations in such neurodegenerative diseases [32,33]. Hence, the cross-subject stability of these rhythm-specific patterns reveals that the proposed method relies on disease-inherent signatures rather than individual-specific noise, directly enhancing its generalizability when data from new subjects are scarce.

Furthermore, the representative channel provides a theoretical solution for single-channel constraints. The FP2 channel identified in this study demonstrates discriminative ability, corresponding to the known neuropathological lesions of AD and FTD [34,35,36]. Even with a single channel, it localizes to cortical areas where disease-specific signals are most concentrated, thereby maximizing entropy and explaining how satisfying accuracy can be maintained with a single-channel input. The prefrontal cortex under neurodegeneration usually reveals altered neural complexity and signal irregularity, specifically the dynamic properties that entropies are designed to quantify. Therefore, the MEFC provides an appropriate level of complexity detail at this location, efficiently capturing the distinct pathological perturbations of executive dysfunction in both disorders into a discriminative feature vector.

Finally, the core contribution of this work is validated not merely by classification, but by the neurobiological plausibility of its entropy features. The method’s applicability under data-limited, cross-subject, and single-channel scenarios stems from its design to capture and fuse stable, pathologically anchored electrophysiological MEFC from informative cortical locations. Such outcomes are beneficial for developing portable diagnostic tools in healthcare applications.

## 5. Conclusions

This study presents a lightweight classification method based on MEFC, specifically designed to address the challenges of data efficiency, cross-subject generalization, and single-channel EEG-based applications in distinguishing AD, FTD, or HC. Particularly, it decomposes EEG data into five classical brain rhythms, extracts and concatenates three complementary entropies (PE, SSE, SE) to form an information-dense feature vector, and employs similarity-based classification. Validated on two public datasets using LOOCV, the MEFC achieves 76.14% three-class (AD, FTD, HC) accuracy and 83.33% two-class (AD, HC) accuracy on the AHEPA dataset and the Florida-Based dataset, respectively. Such performances achieved with an average of a few data points per subject reveal their data-efficient nature. Moreover, the interpretability analysis identifies beta and theta rhythms and the FP2 channel as valuable neurophysiological properties, providing a cross-subject basis for classification in patients with known pathology. Therefore, by integrating a single-channel design with a computationally lightweight entropy-based pipeline, a MATLAB-based toolbox is designed that provides a practical computational solution for developing portable diagnostic tools in biomedical engineering.

While this study validates a promising path for resource-efficient EEG analysis, certain limitations point to future directions. On the one hand, the current feature concatenation approach could be advanced by exploring more sophisticated feature fusion techniques to model the potential interactions between different entropies across rhythms dynamically. Hence, to better characterize complex neural dynamics, future work will investigate integrating the MEFCs with interpretable descriptors within a unified, yet still lightweight, framework, such as recent modules in machine learning fields [37,38,39,40]. On the other hand, another direction is to validate this method for other neurological or psychiatric disorders, such as Parkinson’s disease, depression, and autism, aiming to build a generalizable way for scalable neurodiagnostic screening.

## Figures and Tables

**Table 1:** Key parameters of the experimental dataset assessed in this study.

Parameter	AHEPA	Florida-Based
Classification Task	AD/FTD/HC, three-class	AD/HC, two-class
Number of Subjects	36/23/29	24/24
Number of EEG Channels	19	19
Average Duration	13.5/12.0/13.8 min	8/8 s
Sampling Frequency	500 Hz	128 Hz

**Table 2:** The highest classification accuracies and MADs of five rhythmic MEFCs using the proposed method.

Brain Rhythm	Highest Accuracy (AHEPA/Florida-Based)	MAD (AHEPA/Florida-Based)
Delta (0.5–4 Hz)	52.27%/79.17%	4.65%/8.53%
Theta (4–8 Hz)	64.77%/83.33%	6.21%/8.44%
Alpha (8–13 Hz)	50.00%/64.58%	4.90%/6.25%
Beta (13–30 Hz)	76.14%/72.92%	3.91%/6.14%
Gamma (above 30 Hz)	44.32%/70.83%	4.17%/7.01%

**Table 3:** A comparative study with recent EEG-based works.

Work	Number of Channels	Number of Subjects	Classification Task	Classifier	Accuracy (%)
Shen et al. [11]	19	65	AD vs. HC	3D VGG-inspired CNN	90.77
Tigga et al. [23]	19	18	AD vs. HC	GCN-Transformer	80.73
Zikereya et al. [24]	19	65	AD vs. HC	DAC	83.00
		52	FTD vs. HC		70.00
		59	AD vs. FTD		60.00
Hadiyoso et al. [25]	5	34	AD vs. HC	LDA	82.40
Vo et al. [26]	19	65	AD vs. HC	CNN	80.00
Dharia et al. [27]	6	65	AD vs. HC	DTCA-Net	85.20
		52	FTD vs. HC		67.70
Abdulaal et al. [28]	19	88	AD vs. FTD vs. HC	HFSAN	83.40
		65	AD vs. HC		96.70
Xie et al. [29]	6	88	AD vs. FTD vs. HC	Stockwell-CNN	96.83
This work	1	88	AD vs. FTD vs. HC	MEFC-PCC	76.14
		48	AD vs. HC	MEFC-WC	83.33

## Data Availability

The datasets generated and/or analyzed during the current study are available at https://github.com/LC20251214/AD-and-FTD.git (accessed on 22 December 2025).
